# Cross-Countries Comparison Toward Digital Currency Acceptance: Integrating UTAUT2 Into ITM

**DOI:** 10.3389/fpsyg.2022.944720

**Published:** 2022-07-18

**Authors:** Xin Lin, Qiuxiang Zhang, Din Jong

**Affiliations:** ^1^School of Economics and Management, Northeast Electric Power University, Jilin City, China; ^2^Economic Management Department, Weifang Engineering Vocational College, Weifang, China; ^3^Department of Digital Design and Information Management, Chung Hwa University of Medical Technology, Tainan City, Taiwan

**Keywords:** digital currency, Digital Currency Electronic Payment (DCEP), Extended Unified Theory of Acceptance and Use of Technology (UTAUT2), initial trust factors (ITM), trust, cross-countries comparison, usage intention, partial least squares

## Abstract

In the context of digital monetary market integration, the importance of cross-border digital currency research is receiving prominent attention. This study integrated Extended Unified Theory of Acceptance and Use of Technology (UTAUT2) and initial trust factors (ITM) into an integrative framework, which synthetically complemented the objective measures and subjective insights of digital currencies. The results indicated the integrated framework, which verified its robustness predicting the acceptance and recommendation intention of digital currency. By analyzing the two different features of digital currencies, this research puts forward a set of targeted solutions to ensure that users of Chinese and Korean digital currencies make a long-term policy for the sustainability, eventually benefitting the cross-border digital monetary transactions and economic cooperation in Asia, which leads the world to the sustainable development in the digital currencies field.

## Introduction

Nowadays, digital currency is still in its infancy, but it has the potential to fundamentally alter the balance between the mobile payments, business banks, central banks, and the traditional economic power. With the maturity of the digital currency and the rapid development of related digital technologies, as a revolution payment method, mobile payment has quietly triggered a payment innovative all over the world (Li et al., 2019). The academics can derive the future of China's development of the world's leading digital payment infrastructure. By 2025, mobile payments are worth about 75% of GDP, nearly twice the amount in 2012. Currently, <50% of China's business transactions are completed through digital payment, far more than the level of developed marketplaces (Reid and Marion, 2020). In the end of 2019, the Chinese government announced the launch of the Digital Currency Electronic Payment (DCEP) without a formal statement. The plan was finalized and approved in October 2019, so it seems feasible to pilot it by the end of 2020. China would be the first to promote the circulation of DCEP if all was achieved as scheduled. It will sustainably stimulate other major economies to create other specific digital currencies. The digital currency issued by the central banks may become a powerful political and economic means for China, which is developing DCEP supported by the central bank as an executive power tool. Indeed, if enterprises conducting business in China were required to accept the DCEP, it would surely maintain global monetary markets' stability and promote global economic growth (Shi and Zhou, 2020).

Digital currency, whether issued privately or publicly, is a currency expressed in some digital forms, such as, digital currency can be centralized with one center controlling the currency supply (i.e., China's DCEP), or decentralized with coming from various sources (i.e., Japan's virtual currency, Korea's digital currency). The country with the global largest digital currency users is South Korea (Dahlberg, 2019). In March 2020, Korea passed the world's first comprehensive digital currency law, named “Specific Financial Information Act.” As South Korea's most popular carrier apps (Kakao Corp.), KakaoTalk and KakaoPay subsidiary has 90% permeability in South Korea's messaging and fintech markets. Kakao Corp. also has a plan to create a global public digital currency named Klaytn, developed by Ground X, a crypto monetary exchange operated by South Korean fintech company Dunamu, in Singapore and Indonesia. In addition, Kakao Corp. has launched a popular Upbit encryption currency in South Korea for the encryption currency. Since 26 September 2019, Klaytn has been designed specifically for the Asian clients, such as Indonesia, and has addressed the unique features of the Asia area's users, which are seen as trying to dig up the country's vast, unbanked population, according to the South Korean Internet company Kakao. As an updated version to existing monetary services, digital currency makes use of blockchain technology, which is expected to offer significant sustainable profits to the consumers. To confirm the influencing factors to the acceptance of digital currencies, the usefulness and advantages of digital currencies must be recognized (Silinskyte, 2014; Kumpajaya and Dhewanto, 2015). Therefore, Ermakova et al. (2016) listed multiple benefits of digital currencies, for instance, improved anonymous cashless m-payments' willingness, decreased transaction costs, global use, and increased opportunity to gain digital currency.

Judged by the above literature review, as both China and Korea proposed electronic, cryptographic, and P2P strategies, the focus of international economic development is likely to shift (Reid and Marion, 2020). There are a few studies on usage intention of the digital currency payment (Guych et al., 2018). There has even been no research available on the influencing factors of digital currency payment between China and Korea users' adoption willingness. Because China's DCEP and South Korea's Klaytn are in the trial operation stage, it is very urgent to study the influencing factors of their usage intention. It has a certain theoretical contribution and practical value for this study to fill this gap.

This research provides two innovations as follows. First, most previous studies regarded users as an integral sample, and a few academic studies explored the regulatory role of different digital currencies' characteristics in the process of building trust in the monetary market (Akturan and Tezcan, 2012; Afshan and Sharif, 2016; Baabdullah et al., 2019). There are currently very few studies that can integrate ITM into UTAUT2 to form an integrated framework to test the factors that influence the willingness to use mobile payments. Furthermore, on the one hand, UTAUT2 systematically revealed the objective technical characteristics of performance expectancy, effort expectancy, user satisfaction, social influence, facilitating conditions, hedonic motivation, price value, and habit. On the other hand, UTAUT's limitation is lack of evaluation to personal propensity to trust, structural assurance, firm reputation from the adoption willingness. In this research, ITM compensated the UTAUT's lack of analyzing trust series factors about digital currency, which could positively influence the usage intention by the immediate element of usage satisfaction in the core construct of UTAUT. The defects of two frameworks were jointly compensated by integration. The first purpose of this research in considering the combined impact of different digital currency' initial trust factors (ITM) suggested by Gefen et al. (2003) and Straub and Gefen (2003) technology factors of UTAUT2 proposed by Venkatesh et al. (2012) in the introduction to the concept of an integrated model will help fulfill the existing literature system's gaps.

Second, this study puts forward the comparisons between China's DCEP and South Korea's Klaytn, which will further promote the sustainable development and integration of digital currency technology in Asia, and will also provide a strong driving force for the rapid growth of digital currency economy and technology in the world (Kim and Prabhakar, 2004). In essence, it is very important to confirm the factors that influence the usage willingness of digital currencies and to evaluate the sustainability of the digital currency in different government backgrounds. The second purpose of this research is that if this article can fill the gaps in the literatures of digital currency, it will bring huge commercial benefits.

## Background and Literature Review

### China's DCEP and Korea's Klaytn

Driven by technological developments and a global decline in cash use, many central Banks are exploring the possibility of issuing digital currencies to replenish cash (PricewaterhouseCoopers, 2019). DCEP is a controllable and anonymous payment instrument issued by the people's Bank of China, which is operated by the designated operating agencies and converted to the public. It is based on the generalized account system, supports the loose coupling function of bank accounts, is equivalent to banknotes and coins, and has value characteristics and legal compensation. Like an entity's fiat currency or cash, DCEP owns all the national currency's features (Cooper et al., 2019), which can be used for daily payment or as a store of value. On the one hand, the China's DCEP is a latest highly decentralized virtual currency. It is a P2P technology that uses a process called cryptography, in which common information is converted into passwords or incomprehensible text to ensure transaction safety, enlarge currency supplement, and avoid fraud (Kim, 2016). The China's DCEP will directly describe an instantaneous picture of monetary activity in a major economy or area, and it can also provide more precise China's GDP estimates' data than those currently available (PricewaterhouseCoopers, 2019).

On the other hand, Korea cross-border business transactions can be conducted by using Klaytn coin without the involvement of the central bank's intermediary (Dahlberg, 2019). The transaction's instantaneous features offered by the Korea's Klaytn such as middlemen removal (Ermakova et al., 2016; Walton and Johnston, 2018), shorter transaction time (Baur et al., 2015; Gunawan and Novendra, 2017), and lower cross-region cost (Baur et al., 2015; Ermakova et al., 2016) made Klaytn acceptable and promised to bring benefits to the users. Besides the representativeness of digital currency (e.g., Klaytn), Korea owns hundreds of sustainable alternatives with dissimilar advantages. Irreplaceable and innovative digital currency characteristics, including but not limited to increase transaction speed (Gao et al., 2016; Gunawan and Novendra, 2017; Mendoza-Tello et al., 2018), reduce business deal costs of remittances (Ermakova et al., 2016; Folkinshteyn and Lennon, 2017), namelessness, and privacy (Shehhi et al., 2014; Seetharaman et al., 2017), and the removal of intermediaries (Gao et al., 2016; Walton and Johnston, 2018).

Overall, China's DCEP and Korea's Klaytn is a potential regulated alternative to private digital currencies. Furthermore, compared with Klaytn, DCEP is considered to be a better supplement or substitute for physical cash (Raskin and Yermack, 2016). For Klaytn in a digital form, DCEP represents a more legal payment method and is generally accepted as legal tender supported and regulated by the global central bank (Cooper et al., 2019).

### Unified Theory of Acceptance and Use of Technology

According to the previous literatures, TAM theory is frequently applied in various studies and can only describe 40% of the usage intentions (Mendoza et al., 2017), while the UTAUT model accounts for over 70% of the usage willingness (Schaper and Pervan, 2007). Obviously, it is clear that UTAUT owns more realistic statistical significance and more explanatory power. Almost at the same time, Venkatesh et al. (2012) expanded UTAUT with price value, hedonic motivation, and habit, and finally proposed UTAUT2, which further improved the interpretation ability of UTAUT model.

UTAUT2 model is widely applied to examine and confirm the factors influencing the intention to accept mobile business (Chopdar et al., 2018; Shaw and Sergueeva, 2019), mobile transactions (Farah et al., 2018; Gyu, 2019), and mobile banking (Khan et al., 2017; Lee and Han, 2019). Obviously, the recent studies incline to integrate various theoretical structures to gain a more comprehensive perspective in researching a new technology users' willingness (Lee and Mu, 2017; Sung, 2019). This research combines UTAUT2 with ITM as a comprehensive integrated framework to assess the elements influencing usage intention to digital currencies between China and Korea. Integrating UTAUT2 with ITM revealed the necessity to fill the theoretical gaps in the area of monetary information technology (Tam and Oliveira, 2017).

### Initial Trust Model

Initial trust model (ITM) was termed as “the intent to use trust by customers to meet requirements in the absence of experience or reliable, in-depth information (McKnight et al., 2002; Kim and Prabhakar, 2004).” Thus, Kim et al. (2009) built ITM, in which the initial trust of m-banking could be explained by structural assurance, personal propensity to trust, and firm reputation. ITM theory has been studied in many studies, for instance, m-banking (Martins et al., 2014), m-payment platforms (Chandra et al., 2010), m-shopping (Lu and Su, 2009), online banking (Fisher et al., 2008), and m-healthcare (Fisher et al., 2008).

This research applies the trust theory in e-commerce to digital currency's study: ITM is confirmed to be effective after testing in various business environments; however, the importance of every construct will change differently, which requires some extensions. Particularly, a lot of differences exist in the role of initial trust in digital transactions, which is necessarily integrated into a new transaction trust framework supported by digital currency systems, with a new construct such as UTAUT2 (Zarifis et al., 2014).

In other words, under the broader background of digital currency, it is necessary to discuss the positive correlation between users' willingness to adopt digital currency and the level of digital currency reputation, users' propensity to trust, and the degree of government regulation improvement. Thus, it is ensured that these three supplementary parameters are augmented to ITM to create a user trust model that supports digital currency transactions (Zarifis et al., 2014).

## Research Model

Performance expectation (PE) means the degree that users think the adoption of relevant digital currency will contribute to their work performance (Venkatesh et al., 2003). As mentioned earlier, digital currency uses blockchain technology functions with unique business function to solve the traditional monetary services' shortcomings. Therefore, users can obtain significant benefits from adopting digital currency. Kumpajaya and Dhewanto (2015) and Shahzad et al. (2018) believed that the recognition of digital currencies in daily transactions was very important in acceptance of digital currencies. Judged by the existing literatures, if they trust that the benefits of using digital currency outweigh the losses, the digital technology will be more likely to be adopted (Venkatesh et al., 2012). Besides, PE was pointed out to be the most influential and discernable determining factor in a new technology's usage intention (Venkatesh et al., 2003).

The existing empirical studies on m-payment acceptance (Kraljić and Pestek, 2016) and m-banking acceptance (Connolly and Kick, 2015; Ermakova et al., 2016) revealed that usage intention is positively affected by PE. Many previous studies (Chong, 2013; Faqih and Jaradat, 2015) clearly support the positive impact of m-payment adaption willingness. Obviously, PE is a key factor in the user evaluation process. So, the following hypothesis is suggested:

H1: Performance expectancy significantly influences user satisfaction.

Effort expectancy (EE) means the degree that an individual can use a m-payment system effortlessly (Venkatesh et al., 2003). User will start judging whether their mobile wallet is easy from the registration process. Users can comfortably accept a digital currency when conducting m-commerce business.

Digital currency is still in its infancy as a new paradigm in fintech (Nakamoto, 2008). Kumpajaya and Dhewanto (2015) and Henry et al. (2018) have shown the acceptance of digital currencies verified by a personal belief in the perceived ease degree to digital currency. It is expected that the necessary knowledge to use digital currency will be outstanding (Kumpajaya and Dhewanto, 2015). From a financial viewpoint, users should own basic financial knowledge to monitor price value in avoiding unexpected high monetary costs. In the technical viewpoint, digital currency users have the responsibility to protect their digital wallet, which are vulnerable to security attacks and m-payment process is irreversible. Yu (2012) and Slade et al. (2015) indicated that EE positively influences the behavioral intention. The following hypothesis is suggested:

H2: Effort expectancy significantly influences user satisfaction.

Social influence (SI) suggests that if users' close social support powers (such as family, friends, or leaders) have confidence in a new digital currency, then it would be definitely accepted (Venkatesh et al., 2012; Tam and Oliveira, 2016). Particularly when the current user plans to thoroughly alter from using one digital currency to using another, his usage intention will be easily affected by peers, family relatives, friends, etc. (Baptista and Oliveira, 2015; Dwivedi et al., 2017). Existing studies have revealed that SI played an important element affecting users' usage intention of m-banking (Slade et al., 2015) and m-payment (Yu, 2012).

Given that money is a social and psychological product, SI is anticipated to be an important driver digital currency adoption. In the context of digital technology dominating the network world, the influence of SI will not only impact the willingness of using an old technology, but also affect users to turn to a new digital technology recognized by SI (Al-Somali et al., 2009; Williams et al., 2015). The following hypothesis is suggested:

H3: Social influence significantly influences user satisfaction.

Facilitating conditions (FC) means the extent that a consumer trusts whether the existing infrastructure will facilitate him to adopt a digital system (Thompson et al., 1991; Venkatesh et al., 2003). Existing empirical studies on the usage willingness of different digital technology indicated that usage intention is positively affected by FC (Yu, 2012; Slade et al., 2015).

Regulations formulated by regulators play a vital role in public acceptance (Shahzad et al., 2018). So, this perspective may enhance technological infrastructure's development to support the digital currency-based payment systems. According to Queiroz and Wamba (2019), FC influences digital usage adoption in the developed countries (e.g., Canada and USA), but it has no effect in the context of a developing country (for instance, India). Conversely, Gunawan and Novendra (2017) pointed out that FC influences the Indonesia users' adoption of digital currency, who are satisfied with the facility conditions and organized foundation of the digital currency. The following hypothesis is suggested:

H4: Facilitating conditions significantly influences user satisfaction.

Hedonic motivation (HM) means the degree of satisfaction in adopting m-banking's progression (Venkatesh et al., 2012), which is an important pre-testing element influencing users' willingness of adopting a new technology (Heijden, 2004). Many existing studies have shown that HM has a positively influence in predicting the willingness to use various digital currency application scenarios (Alalwan et al., 2017; Herero et al., 2017). Venkatesh et al. (2012) pointed out that HM was the second major element influencing the willingness to adopt a new technology.

If a new digital currency payment technology provided sufficient comfort, satisfaction, and entertainment, users will not transfer their intention of use to other competitive payment services (Koenig-Lewis et al., 2010; Alalwan et al., 2015; Baabdullah, 2018). First, an individual takes the choice of using digital currency for investment purposes to give you a sense of satisfaction when investing return to build. Obviously, the joy and happiness brought by the income of digital currency investment positively influences the user's behavior intention. The second intrinsic motivation offered by digital currency is the freedom feeling by using anonymous digital currency technology, which will motivate users to participate in the forwarding of the digital currency (Bashir et al., 2016). Third, the attraction of individuals to novel digital currency will also increase HM. Accordingly, the following hypothesis is submitted:

H5: Hedonic motivation significantly influences user satisfaction.

Price value (PV) refers to an appreciable balance between the digital currency service profits of users' experience and the monetary costs in using digital currency services of users' experience (Venkatesh et al., 2012), including the cost of digital currency service, equipment cost, and after-sales cost. When the profits outweigh the costs, users are more inclined to choose a specific technology as well as a mobile platform (Yu, 2012; Slade et al., 2015). After UTAUT2 extended with PV, some studies (Rondan-Cataluna et al., 2015; Alalwan et al., 2017; Lallmahomed et al., 2017) have revealed that PV positively influences the usage intention.

Considering that the digital currency's price is limited by demand and supply in the global transition market, digital currency market is inevitably highly volatile compared with fiat currency (Berentsen and Schär, 2018). As a result, the sharp increase in price volatility has attracted users' intention of its acceptance (Farell, 2015; Henry et al., 2018). The value of a particular digital currency unit comes from the users' trade-off between the benefits of using digital currency and the relative financial costs (Yeong et al., 2019). Given the above situation, the following hypothesis is suggested:

H6: Price value significantly influences user satisfaction.

Habit (HA) means the degree that an individual tends to carry out as a result of learning (Limayem et al., 2007; Venkatesh et al., 2012). Unlike experience, HA is coming from previous experiences (Ajzen and Fishbein, 1975, 2005). The empirical conclusions of existing studies revealed that HA positively influences the usage intention of mobile technology, for example, m-banking (Slade et al., 2015) and m-payment (Yu, 2012).

Users are unlikely to change their learned habits and may refuse any unfamiliar communications with m-payment providers (Chemingui and Lallouna, 2013), because they are inclined to trust acquired experience rather than cognitive reasoning in adopting a new digital currency technology (Venkatesh et al., 2016; Zhang et al., 2017). As a new type of digital innovation in the monetary technology, the extent to which users automatically exchange goods and services using traditional legal tender in their daily lives affects their willingness to adopt digital currency. Under the background of digital payment, following the structure of UTAUT2, habit significantly influences the usage intention of a new technology. Accordingly, the following hypothesis is suggested:

H7: Habit significantly influences user satisfaction.

As a vital construct of ITM, structural assurance (SA) means the perceived lawful and technical protection of specific users (Mahfuz et al., 2016) and its impact on initial trust. According to the abovementioned viewpoints, it can be easily concluded that in the process of digital currency payment transactions, the people can feel the initial trust under the dual roles of relevant government institutions, industry rules, social supervision, contracts, etc. In monetary business, structural assurance is more urgent (Kim and Prabhakar, 2004). As outcomes from a totally new monetary market, digital currency payment faces multiple risks. Particularly, due to a lack of direct experience, users regarded structural assurance as a vital factor before the adopting digital currency payment, which affects initial trust.

Structural assurance directly affects initial trust and becomes one of the strongest antecedents to initial trust, which can magnify mobile payment's usage intention (Verkijika, 2018). In other words, initial trust improves when users receive structural guarantees from mobile banking (McKnight et al., 1998; Gu et al., 2009). Of course, structural guarantee has been applied to affect initial trust in fashion jewelry (Worasesthaphong, 2015), e-commerce (Xin et al., 2015; Alqatan et al., 2016), and mobile banking (Zhou, 2012; Lu et al., 2015; Yu and Asgarkhani, 2015). The following hypothesis is suggested:

H8a: Structural assurance significantly influences initial trust.

Personal propensity to trust is termed as the degree that a personal is inclined to rely on other closed-relationship persons across various situations (McKnight et al., 2002). Personal propensity to trust points out a natural tendency of users toward others. When users accept a higher trust propensity technology, the confidence intensity will sharply increase (McKnight et al., 2002; Zhou, 2011). Personal trust tendency is an attribute characteristic and experience of a person's cultural background and psychological formation (Lee and Turban, 2001). Without prior knowledge, users with a higher level of inclined trust tendency may assume that services are dependable. A lot of studies on the initial trust of m-payment business indicated that the personal trust tendency may positively influence on initial trust in digital currency transactions (Gefen, 2000; McKnight et al., 2002).

In the case of initial trust, personal propensity to trust as a psychological characteristic, from a person's childhood to his or her life entire life, depends on the person's social and cultural background. The research of Wu and Lee (2017) indicated that if safe and accurate m-payment services were provided, the personal propensity to trust will positively influence usage intention. The following hypothesis is suggested:

H8b: Personal propensity to trust significantly influences initial trust.

Corporate reputation refers to the firm's power to provide efficient service to the users and the reliability of users' participation in the firm's transactions (McKnight et al., 1998). A previous study (Bhattacherjee and Sanford, 2006) revealed that reputation of the firm influenced usage intention by the peripheral path. The firm reputation includes the ability to provide services, the reliability of business activities, and the reputation of the enterprise (McKnight et al., 1998). Thus, many famous firms actively provide after-sales service to users, promptly advertise and enhance the firm's high-tech characters, and promote users to trust the famous firm's sufficient technical power and unrivaled competitive advantages, thus greatly improving initial trust of users' business operation platform (Wu and Lee, 2017). The following hypothesis is submitted:

H8c. Firm reputation significantly influences initial trust.

Initial trust refers to the user's willingness to bear unexpected costs to meet demands, without using experience or dependable and referable information (McKnight et al., 1998; Kim and Prabhakar, 2004). Initial trust ensures that users finally achieve the desired results (Pavlou et al., 2003). Particularly under the digital currency payment background, users' trust enhances the individual's performance expectation (Bock et al., 2012). M-payment users can reasonably worry about whether the digital currency platforms can safely transfer and store their own credit card account, password, location privacy, and other privacy information (Martin et al., 2013; Mamonov and Benbunan-Fich, 2015). To increase their life satisfaction degree and work performance, only a high-quality digital currency service platform can meet their expected needs (Zhou, 2011). Therefore, in the absence or lack of experience, initial trust plays a crucial role in choosing an updated technology services, for instance, digital currency services (Kim and Prabhakar, 2004; Kim et al., 2009). Therefore, this article describes that initial trust may affect sustainability of digital currency transactions, hence makes the following hypotheses:

H9a: Initial trust significantly influences performance expectancy.
H9b: Initial trust significantly influences user satisfaction.
H9c: Initial trust significantly influences usage intention.
H10: User satisfaction significantly influences usage intention.

**Figure 1:**
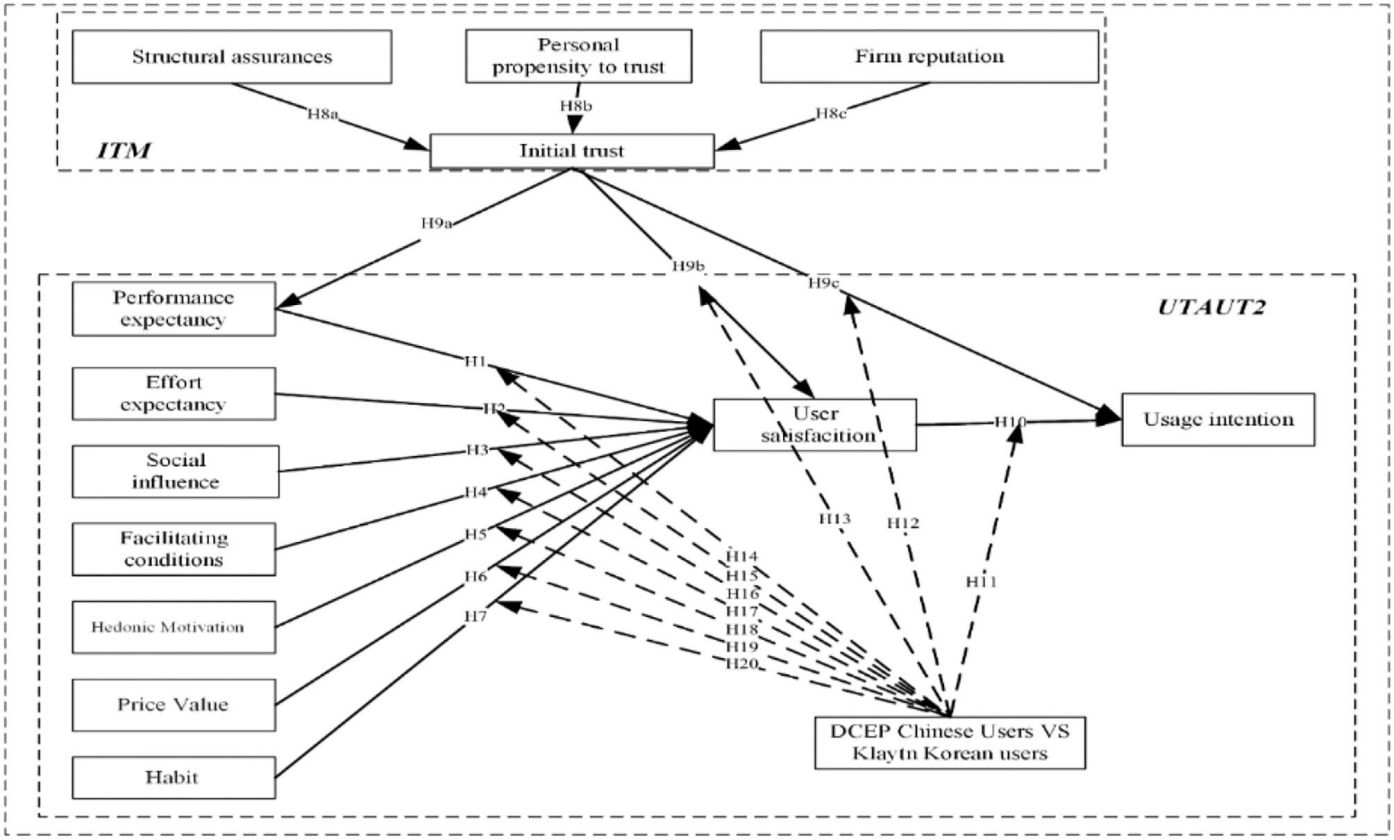
Research model.

According to the development of above hypotheses, this article proposed our research model (as shown in Figure 1).

## Data Collection and Results

### Descriptive Statistics

The original data of this study were collected through a questionnaire survey. This survey adopts statistical analysis method and traditional questionnaire survey to collect information of different groups. It considers issues such as demographics, online payment preferences, awareness and satisfaction with security measures, beliefs and attitudes about the services provided by online payment business providers during the COVID-19 pandemic.

In the following empirical analysis, Cronbach's α was used to calculate the reliability of the measurement means through IBM SPSS 24.0, and the validity of each construct was tested and evaluated by examining factor structure and internal correlation. To test the study hypothesis, we used IBM AMOS 24.0, which also verified the causal relationship between endogenous core variables through standard coefficients and significance values. The entire sample is used to analyze the integrated model prior to the hypothesis verification test. For hypothesis testing, we applied the model to the group for a more specific analysis. Structural equation modeling (SEM) is becoming central to the social sciences and arguably the most popular analytical tool.

Considering all the above hypotheses, this research designed a questionnaire to confirm each model and related measurement component to match the final goal. Furthermore, by modifying every ambiguous detail in our questionnaire, this research facilitated that all respondents completely recognize all points of the questions through one-on-one interviews with potential users coming from the Jilin University in China or the Yonsei University in Korea. A survey of 800 questionnaires were issued and 746 copies were collected (response rate 93.25%). After 94 responses were discarded for a lack of critical data, 652 samples (87.40%) were eventually used for deterministic analysis (321 potential Chinese DCEP users and 331 Klaytn Korean users). The sample chrematistics are summarized in Table 1.

**Table 1:** Sample structure.

**Category**		**Total** **(*****N*** **= 652)**		**China** **(*****N*****1 = 321)**		**Korea** **(*****N1*** **= 331)**	
Gender	Male	396	60.7%	154	47.9%	152	46.02%
	Female	518	54.47%	267	54.93%	251	53.98%
Age	20–30	349	53.5%	160	49.8%	189	57.1%
	30–40	251	38.5%	122	38.0%	129	39.0%
	40–50	35	5.4%	22	6.9%	13	3.9%
	Over 50	17	2.6%	0	0%	17	5.3%
Education	High school student/resident	76	11.7%	48	15%	28	8.5%
	College student/student	327	50.2%	177	55.1%	150	45.3%
	Graduate school or higher	249	38.2%	96	29.9%	153	46.2%
Occupation	Professional	57	8.7%	29	9.0%	28	8.5%
	Self-employed	245	37.6%	116	36.1%	129	39.0%
	Office worker	203	31.1%	103	32.1%	100	30.2%
	Student	130	19.9%	63	19.6%	67	20.2%
	Other	17	2.6%	10	3.1%	7	2.1%

Every element was rated on a five-point Likert scale. For example, the scale ranged from 1, “strongly disagreement” to 5, “strongly agreement.” In the subsequent empirical analysis, IBM SPSS 24.0 was used to figure out the measurement reliability with Cronbach's α. Besides, construct validity was assessed by testing the element structure and internal correlation of every construct. IBM Amos 24.0 was used to examine all these hypotheses by determining the causal relationship among core structures through significance value and standard coefficient. For hypothesis testing, all data were used to analyze the integrated model before the hypothesis testing. Middle-aged customers and women are the largest group using the mobile payment system, with a total of 518 respondents (54.47%) being female, of whom 600 (92%) are suitable respondents aged below 40 years. Table 1 provides detailed statistical results on the characteristics of respondents.

### Reliability, Validity, and Measurement Model Evaluation

Convergent and discriminant validity were applied in the measurement model evaluation, and testing was done by IBM Amos 24.0. According to the results in Table 2, convergent validity refers to the degree to which an evaluation method is associated with others that it should be associated with (Jiang et al., 2013). Three-dimensional indicators were used to measure the convergent validity: (i) the standardized factor loadings, which statistically revealed the correlation between some basic factors and each metric, exceeding the recommended value of 0.50 (Gefen et al., 2003), (ii) the Cronbach's α values were more significant than 0.7 for the reliability of integrated construct (Hair et al., 1998); (iii) the values of AVEs were above 0.50, so the underlying variable explained more than half of the indicator variance (Fornell and Larcker, 1981; Henseler et al., 2009; Hair et al., 2011).

**Table 2:** Convergent validity and reliability (entire samples).

**Construct**	**Indicators**	**Standardized loading**	**Cronbach's α**	**Composite reliability**	**AVE**
US	US 1–4	0.771–0.864	0.882	0.877	0.642
PE	PE 1–4	0.739–0.865	0.913	0.885	0.658
EE	EE 1–4	0.806–0.916	0.902	0.915	0.728
SI	SI 1–4	0.807–0.868	0.903	0.903	0.701
FC	FC 1–4	0.808–0.867	0.895	0.904	0.702
HM	HM 1–4	0.818–0.838	0.875	0.896	0.684
PV	PV 1–4	0.723–0.837	0.883	0.876	0.639
HA	HA 1–4	0.723–0.859	0.882	0.885	0.659
SA	SA 1–4	0.779–0.834	0.871	0.884	0.655
PPT	PPT 1–4	0.772–0.834	0.884	0.875	0.636
FR	FR 1–4	0.788–0.857	0.855	0.887	0.662
IT	IT 1–4	0.728–0.810	0.876	0.862	0.611
UI	UI 1–4	0.749–0.864	0.866	0.869	0.623

*US, user satisfaction; PE, performance expectancy; EE, effort expectancy; SI, social influence; FC, facilitating conditions; HM, hedonic motivation; PV, price value; HA, habit; SA, structural assurances; PPT, personal propensity to trust; FR, firm reputation; IT, initial trust; UI, usage intention*.

One important criterion was used for the evaluation of discriminant validity, i.e., the Fornell-Larcker criterion. Discriminant validity refers to the degree to which items differ between variables (Thong, 2001). Fornell and Larcker (1981) revealed that each AVE's square root should be bigger than each pair of constructs' related correlation coefficient. According to the results of Table 3, each diagonal value (square root of AVE) was bigger than the non-diagonal values (correlations between structures). The cross-loading criterion recommended that the standardized factor loading of each index should be higher than all cross loadings (Fornell and Larcker, 1981).

**Table 3:** Discriminant validity (entire samples).

	**PE**	**EE**	**SI**	**FC**	**HM**	**PV**	**HA**	**SG**	**PTT**	**CR**	**IT**	**US**	**UI**
PE	0.811												
EE	0.367	0.853											
SI	0.404	0.305	0.837										
FC	0.376	0.230	0.372	0.838									
HM	0.376	0.271	0.315	0.285	0.827								
PV	0.366	0.310	0.318	0.267	0.329	0.799							
HA	0.326	0.210	0.333	0.293	0.277	0.299	0.812						
SA	0.406	0.327	0.354	0.282	0.299	0.305	0.276	0.809					
PPT	0.378	0.236	0.278	0.339	0.295	0.272	0.272	0.338	0.797				
FR	0.379	0.235	0.291	0.296	0.224	0.257	0.282	0.227	0.285	0.814			
IT	0.646	0.408	0.472	0.434	0.407	0.430	0.397	0.584	0.601	0.460	0.782		
US	0.602	0.410	0.578	0.537	0.483	0.508	0.476	0.456	0.462	0.391	0.666	0.801	
UI	0.590	0.423	0.464	0.458	0.470	0.478	0.406	0.473	0.445	0.428	0.686	0.714	0.789

*The square roots of AVE are located along the diagonal*.

*US, user satisfaction; PE, performance expectancy; EE, effort expectancy; SI, social influence; FC, facilitating conditions; HM, hedonic motivation; PV, price value; HA, habit; SA, structural assurances; PPT, personal propensity to trust; FR, firm reputation; IT, initial trust; UI, usage intention*.

Judged by the results of the measurement model, this research revealed that the reliability (construct and indicator) and validity (convergent and discriminant) of the constructs are satisfactory. In other words, our measurement methods were proved to have a satisfactory validity.

The measurement and structural models of this study were evaluated by AMOS 24.0. The χ^2^/d.f. are 1.112 and 1.205, GFIs are 0.928 and 0.921, AGFIs are 0.918 and 0.911, NFIs are 0.943 and 0.937, CFIs are 0.994 and 0.989, IFIs are 0.994 and 0.989, RFIs are 0.937 and 0.932, PGFIs are 0.806 and 0.818, PCFIs are 0.896 and 0.913, PNFIs are 0.851 and 0.865, RMRs are 0.032 and 0.058, and RMSEAs are 0.013 and 0.018. The abovementioned results support for each structure's association.

Structural equation modeling (SEM) tests were carried out for the proposed hypotheses in this research. For the contracted fit index, it exceeds the acceptable fitness minimum, which is a standard value. Each indicator indicated that the fitting results of the examined samples' fitting results confirmed the combined structures were satisfactory.

### Hypothesis Verification

**Figure 2:**
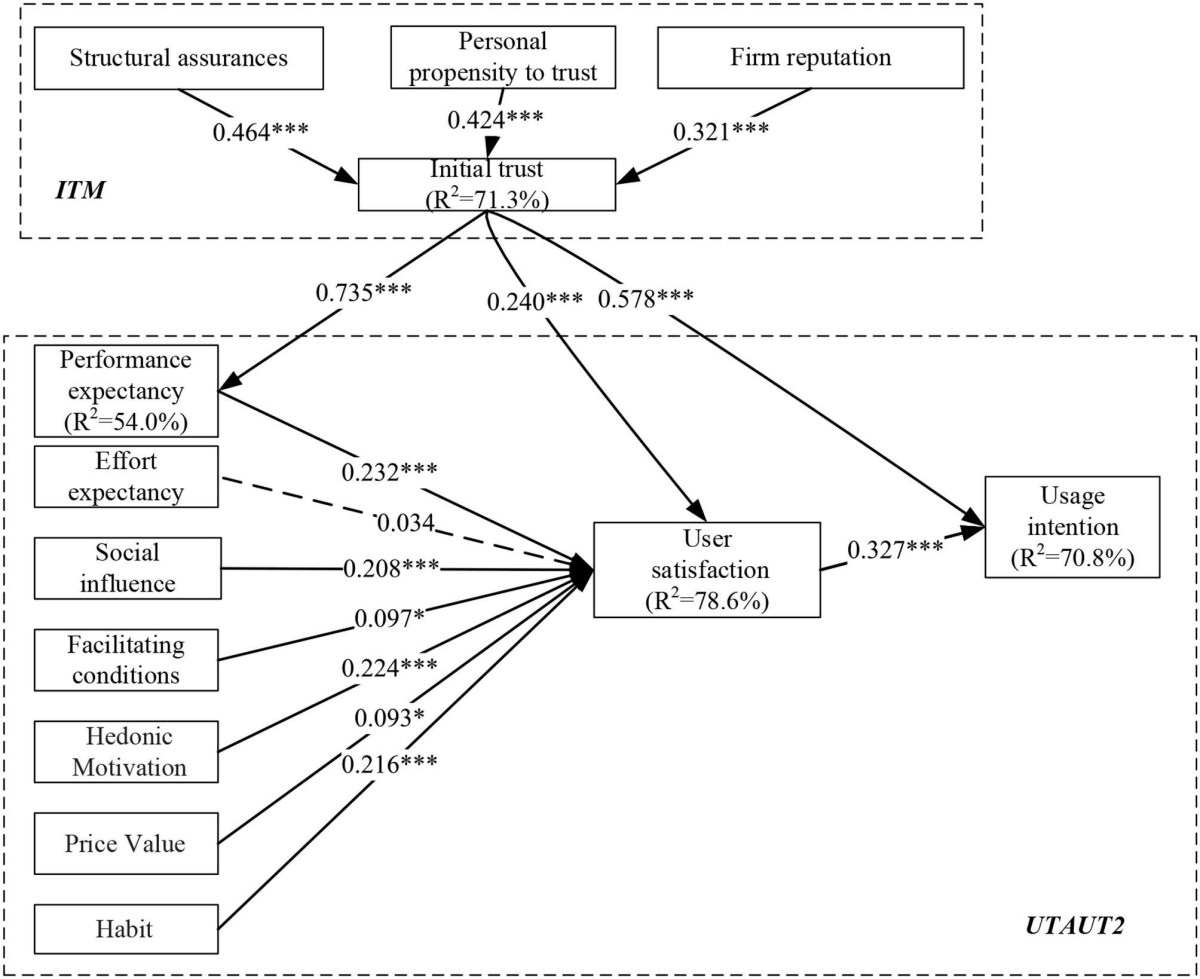
Path analysis of the model (China users). *p < 0.05; **p < 0.01; ***p < 0.001; ns, non-significant.

Next, to examine the organization of this integrated framework, the structure of DCEP's Chinese sample was tested with the Chinese path coefficient, which is revealed in Figure 2. According to the *p*-value, 1 of the 14 paths (H2; *p*-value > 0.05) was rejected, and other 13 paths were proven to be positive.

The DCEP Chinese users' willingness to use generally accounts for 70.8% of the variance usage willingness. The influence on Chinese users (Figure 2) shows that antecedent variables of ITM model and UTAUT2 model account for 71.3 and 78.6% of the variance, respectively, which are related to 70.8% explanatory ability of comprehensive structure on the usage intention.

**Figure 3:**
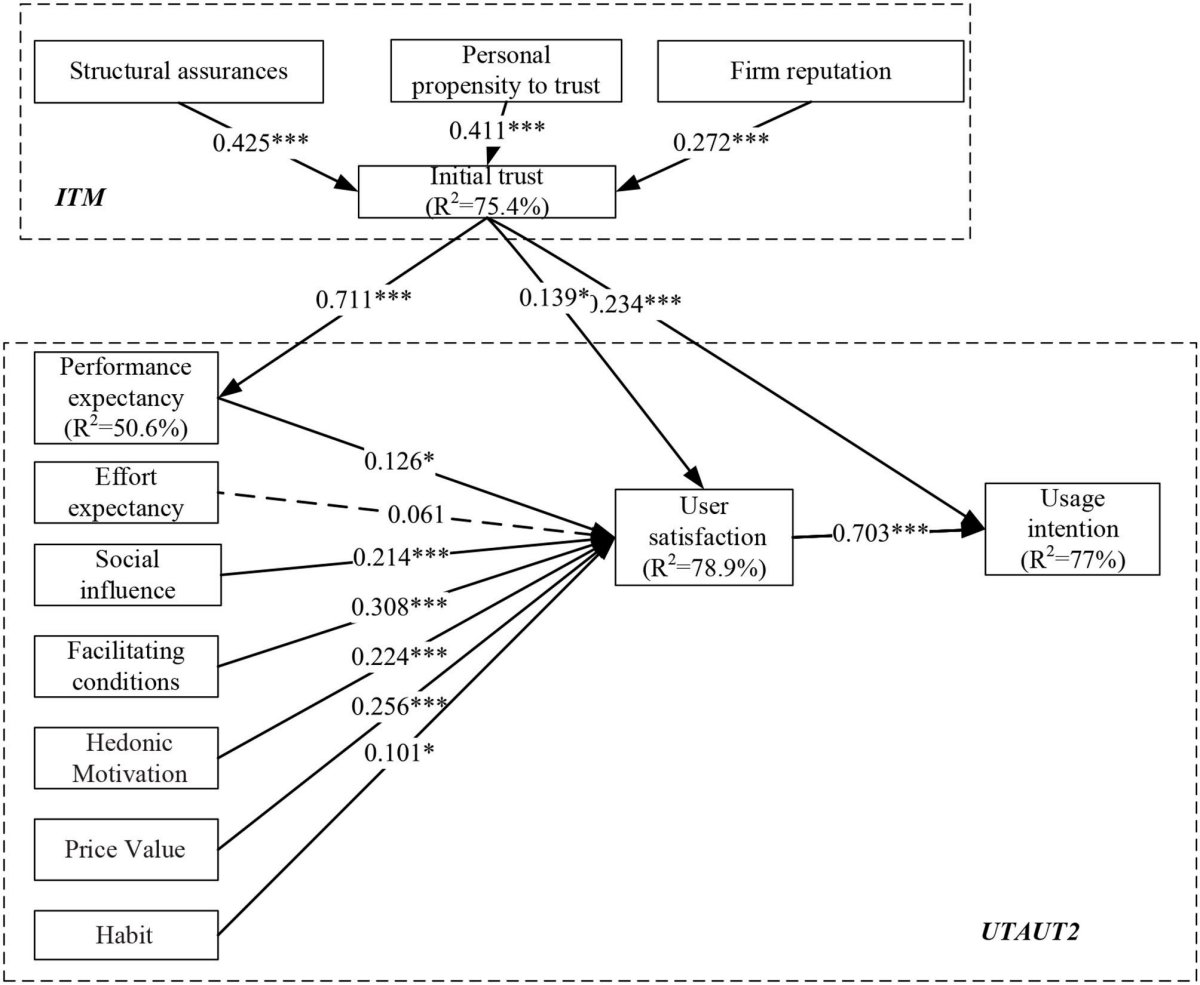
Path analysis of the model (Korea users). *p < 0.05; **p < 0.01; ***p < 0.001; ns, non-significant.

According to the structural results of Figure 3, this research carries out an analysis of the integrated framework of the Klaytn Korea users' sample. Judged by Figure 3, the Klaytn Korean users' pathway coefficient between the basic hypotheses on this research was well evaluated. According to their respective *p*-values, one path of these 14 paths (H2; *p*-value > 0.05) failed, and the rest of the paths were statistically positive.

Klaytn Korea users' willingness to use generally accounts for 77.1% of the variance usage intention. The influence of DCEP China users (Figure 3) shows that prerequisites of ITM and UTAUT2 can explain the variance of 75.4 and 78.9%, respectively, which are related to 77.1% explanatory ability of comprehensive structure on the usage intention.

Table 4 reveals the characteristics of the variables, including the combined framework's coefficients and the hypothesis-analyzed results. According to their respective *p*-values, one of the 14 pathways (H2; *p*-value > 0.05) was not supported, and the other 13 pathways were significantly below the 0.05 level.

**Table 4:** Results of hypotheses tests (entire samples).

**Hypothesis**	**Route**	**Path coefficients**	**T-value**	**P-value**
H1	PE→US	0.174	4.117	^***^
H2	EE→US	0.057	1.937	ns
H3	SI→US	0.219	6.595	^***^
H4	FC→US	0.200	6.345	^***^
H5	HM→US	0.149	4.814	^***^
H6	PV→US	0.179	5.522	^***^
H7	HA→US	0.147	4.773	^***^
H8a	SA→IT	0.443	12.069	^***^
H8b	PPT→IT	0.416	11.168	^***^
H8c	FR→IT	0.299	8.969	^***^
H9a	IT→PE	0.726	14.563	^***^
H9b	IT→US	0.197	4.081	^***^
H9c	IT→UI	0.378	7.384	^***^
H10	US→UI	0.549	10.811	^***^

*** *p < 0.001; ns, non-significant; US, user satisfaction; PE, performance expectancy; EE, effort expectancy; SI, social influence; FC, facilitating conditions; HM, price value; HA, habit; SA, structural assurances; PPT, personal propensity to trust; FR, firm reputation; IT, initial trust; UI, usage intention*.

The comprehensive structure was examined by Korean samples (Table 5), showing that the integrated model was supported. According to the results, one of the 14 paths (H2; *p*-value > 0.05) was not supported, and the rest of the paths were significantly below the 0.05 level. The comprehensive model was analyzed with the Klaytn Korea users' samples (Table 5).

**Table 5:** Results of hypotheses tests (Korea users' samples).

**Hypothesis**	**Route**	**Path coefficients**	**T-value**	***P*** **-value**
H1	PE→US	0.126	2.316	^*^
H2	EE→US	0.061	1.529	ns
H3	SI→US	0.214	4.728	^***^
H4	FC→US	0.308	6.852	^***^
H5	HM→US	0.224	4.696	^***^
H6	PV→US	0.256	5.527	^***^
H7	HA→US	0.101	2.460	^*^
H8a	SA→IT	0.425	8.338	^***^
H8b	PPT→IT	0.411	7.439	^***^
H8c	FR→IT	0.272	5.550	^***^
H9a	IT→PE	0.711	10.502	^***^
H9b	IT→US	0.139	2.156	^***^
H9c	IT→UI	0.234	3.637	^***^
H10	US→UI	0.703	10.036	^***^

* *p < 0.05;*

*** *p < 0.001; ns, non-significant; US, user satisfaction; PE, performance expectancy; EE, effort expectancy; SI, social influence; FC, facilitating conditions; HM, hedonic motivation; PV, price value; HA, habit; SA, structural assurances; PPT, personal propensity to trust; FR, firm reputation; IT, initial trust; UI, usage intention*.

Table 6 shows normalized coefficients of the pathways, the causal path characteristics, and the confirmed hypothesis results. The empirical analysis results of DCEP China users are shown in Table 6, which confirmed the existence of the comprehensive model. Taking DCEP China users' samples as an example, one of the 14 paths (H2; *p*-value > 0.05) were not supported, and the rest of the 13 paths were significantly below 0.05.

**Table 6:** Results of hypotheses tests (China users' samples).

**Hypothesis**	**Route**	**Path coefficients**	**T-value**	***P*** **-value**
H1	PE→US	0.232	3.519	^***^
H2	EE→US	0.034	0.795	ns
H3	SI→US	0.208	4.330	^***^
H4	FC→US	0.097	2.178	^*^
H5	HM→US	0.224	4.696	^***^
H6	PV→US	0.093	2.015	^*^
H7	HA→US	0.216	4.621	^***^
H8a	SA→IT	0.464	8.601	^***^
H8b	PPT→IT	0.424	7.982	^***^
H8c	FR→IT	0.321	6.706	^***^
H9a	IT→PE	0.735	10.002	^***^
H9b	IT→US	0.240	3.327	^***^
H9c	IT→UI	0.578	7.031	^***^
H10	US→UI	0.327	4.521	^***^

* *p < 0.05;*

*** *p < 0.001; ns, non-significant; US, user satisfaction; PE, performance expectancy; EE, effort expectancy; SI, social influence; FC, facilitating conditions; HM, hedonic motivation; PV, price value; HA, habit; SA, structural assurances; PPT, personal propensity to trust; FR, firm reputation; IT, initial trust; UI, usage intention*.

### The Differences Analysis of Path Coefficients Between DCEP and Klaytn Samples

The empirical results of hypothesis regulation (H11–H20) are shown in Table 7. First of all, judged by the *p*-values of SI and EE on usage intention, the moderating effect of DCEP and Klaytn groups is not significant. Second, the rest of seven *p*-values show differences in the regulatory effect between DCEP and Klaytn groups. In the DCEP's Chinese users' group, PE (β = 0.232, *p* < 0.01), SI (β = 0.208, *p* < 0.01), HM (β = 0.224, *p* < 0.01), HA (β = 0.216, *p* < 0.01), and IT (β = 0.240, *p* < 0.01) positively influence the usage intention at 5% basic level, unlike in the Klaytn's Korean group. In contrast, SI (β = 0.214, *p* < 0.01), FC (β = 0.308, *p* < 0.01), and PV (β = 0.256, *p* < 0.01) positively affected the basic level of usage intention at 1% in Klaytn's Korean user group, unlike in the Chinese travelers' group. The consequence of this research showed the significance of country moderators in strengthening the explanation power to digital currencies' usage intention in the UTAUT2-based integrated model.

**Table 7:** The path coefficients' difference between China and Korea users.

**Route**	**DCEP**		**Klaytn**		**Pairwise parameter comparisons**	
	**β**	***P*** **-value**	**β**	***P*** **-value**	***T*** **-value**	***P*** **-value**
US→UI	0.327	^***^	0.703	^***^	3.388	0.001
IT→UI	0.578	^***^	0.234	^***^	−3.488	0.001
IT→US	0.240	^***^	0.139	0.031^*^	−1.027	0.305
PE→US	0.232	^***^	0.126	0.021^*^	−1.183	0.238
EE→US	0.034	0.426	0.061	0.126	0.497	0.620
SI→US	0.208	^***^	0.214	^***^	0.293	0.770
FC→US	0.097	0.029^*^	0.308	^***^	3.003	0.003
HM→US	0.224	^***^	0.104	0.012^*^	−2.102	0.036
PV→US	0.093	0.044^*^	0.256	^***^	2.519	0.012
HA→US	0.216	^***^	0.101	0.014^*^	−2.004	0.046

* *p < 0.05;*

*** *p < 0.001; ns, non-significant; US, user satisfaction; PE, performance expectancy; EE, effort expectancy; SI, social influence; FC, facilitating conditions; HM, hedonic motivation; PV, price value; HA, habit; SA, structural assurances; PPT, personal propensity to trust; FR, firm reputation; IT, initial trust; UI, usage intention*.

## Conclusion

### Discussion

With the rising popularity of DCEP and Klaytn, the digital currencies are expected to gain rapid prominence. In order to confirm the promoters and inhibitors of m-payment, as well as users' response to digital currencies, this study developed a new integrated framework that combines UTAUT2 with ITM, as well as perceived technological security and recommendation intention. The results of this research pointed out that the integrated framework showed better explanatory power in describing consumers' willingness to choose digital currencies as a trustful technology.

On the one hand, from the perspective of ITM model, personal propensity to trust (Gefen et al., 2003) is considered to be the user personality that affects initial trust (Gefen et al., 2003) and is related to user satisfaction, which means to increase the personal trust, tendency can positively influence the initial trust, just like firm reputation and structural assurances (McKnight et al., 2004; Kim et al., 2009). In other words, firm reputation and structural assurances, which refer to company image and security system scale are also the trust's affected indicators, and both have a positive impact on initial trust (Flavian et al., 2005; Chen and Barnes, 2007; Fuller et al., 2007). This research examined personal trust tendency, structural assurances, and firm reputation because all these variables were used by Kim et al. (2009) in their ITM model. According to Gefen et al. (2003), users will obtain positive results in the future through initial trust because initial trust can rise the performance expectancy, increase user satisfaction, and positively influence on buying willingness (Pavlou and David, 2004; Pavlou and Fygenson, 2006; Chang and Chen, 2008).

On the other hand, from the perspective of UTAUT2 model, the findings of both DCEP and Klaytn data revealed that effort expectancy (H2) was not a significant indicator of the willingness of adopting digital currency. However, the results confirmed the significance of PE (H1), SI (H3), FC (H4), HM (H5), PV (H6), and HA (H7) on the willingness of adopting digital currency.

In general, in the context of the exponential growth of digital currency, a comprehensive and comparative perspective is beneficial to other studies, by increasing the possibility of influencing factors to the global digital currencies. Due to the extensive cooperation between China and South Korea in the digital currency area from early 2019, by comparing the differences between users of two countries, the core factors influencing the willingness of consumers to use were stimulated, so as to provide the necessary theoretical basis and practical preparation for the sustainable mobile payment market cooperation between China and South Korea on a larger scale.

### Theoretical Contribution

Generally, the theoretical contributions of this research are three-fold and are illustrated as follows. First, few scholars have focused on the usage intention of potential users to choose either DCEP or Klaytn. A research blank exists in the digital currency's technology that was not examined at all. This comparison method improved the efficiency of some specific scenarios examining between China and Korea, which also fills the above blank in the digital currency's research. Furthermore, this research also tested the moderating variables and multigroup analysis of the DCEP and Klaytn dissimilarities by improving the integrated multi-model method. The blank of digital currencies' research was increased by testing the adjustment variables under the Chinese and Korean different regulators.

Second, this study proposed a more comprehensive integrated framework that was combined with ITM (Kim and Prabhakar, 2004) and UTAUT2 (Venkatesh et al., 2012) to confirm influencing factors that facilitated the initial trust, while examining the technology of the user terminal characteristics and the initial trust's influence on usage intention. According to the results of this research, it is concluded that the combined model provides a stronger interpretation on adoption willingness than UTAUT2 and ITM, separately, which is consistent with previous studies (Lin et al., 2019, 2022; Lin and Wu, 2021; Lin X. C. et al., 2021; Lin X. et al., 2021). Compared with the single-model analysis, this two-dimensional combined framework can comprehensively confirm the factors that positively influence the usage intention of digital currency's technology.

Third, this research contributed to extend UTAUT2 with ITM, and also explain the moderating power of user satisfaction. As an important crossing-point, the digital currency user's satisfaction can be the essential precondition factor. Inspired by this research's attempt to extend UTAUT2 in a multi-model and multi-group integration viewpoint, studies of future digital currency may consider a more systematical perspective to examine the usage intention of any digital currency.

### Managerial Implications

From a managerial perspective, the managerial implications of this research are proposed as follows. First, this study tested the effect of initial trust and digital technology variables on usage adoption of digital currency users. This evaluation process revealed the effect of several comparison scenarios between DCEP Chinese users and Klaytn Korean users to point out the specific new blanks in the research of digital currency. The final goal is to examine the existing UTAUT2 model with different digital currencies rather than the DCEP and Klaytn of this research to improve the Asia and global monetary markets' development. Thus, the results of this research have the managerial implication to both the academics and the digital currency's providers. The practitioners can plan, improve, and introduce the integrated framework in digital currency's perspective to influence the usage willingness of the consumers.

Second, the findings of this study suggested that the digital currencies' providers should generate the initial trust among users to facilitate their usage intention. If the digital currency turns into real currency, it will consider two major problems, namely, (i) the government policies, the related laws, and regulations to digital currency are important elements positively influencing the future usage intention of digital currency. Selecting the accessible, revolution, and guideline or traditional, limited, and forbidden will strongly influence the acceptance and spread of the digital currency in the global countries. (ii) Even in a moderate market environment, because of the security and convenience of the digital currency business with technical threshold, it is still rather high for some ordinary users. Therefore, the widespread adoption of digital monetary market still requires technological and application innovation, as well as the follow-up of relevant laws and regulations.

### Limitations and Future Work

Although our research owns a few contributions in theory and management, it also has other limitations and deserves further research; the future research direction remains to be explored. Understanding the relationship between DCEP and other digital currencies in the global different countries is another interesting area to explore. Due to the different maturity and usage of digital currencies, research across multiple countries can offer additional perceptiveness (Dennehy and Sammon, 2015). In addition, security is the last important direction of future study. As so complex production, digital currency transcends the boundary of digital technology, including the dependability of business parties, where dataset is safely settled (device or the digital cloud), proprietorship of the business dataset, and regulatory and administration culture background, including privacy lawmaking. It is worthy of discovering these issues jointly or separately.

## Data Availability Statement

The raw data supporting the conclusions of this article will be made available by the authors, without undue reservation.

## Ethics Statement

Ethical review and approval was not required for the study on human participants in accordance with the local legislation and institutional requirements. Written informed consent for participation was not required for this study in accordance with the national legislation and the institutional requirements.

## Author Contributions

Conceptualization, methodology, writing—original draft, and writing—review and editing: XL, QZ, and DJ. Formal analysis and investigation: XL. Visualization: QZ and DJ. Validation: DJ. All authors contributed to the article and approved the submitted version.

## Funding

This research was funded by Doctoral Research Initiation Fund Program of Northeast Electric Power University (BSJXM2020214).

## Conflict of Interest

The authors declare that the research was conducted in the absence of any commercial or financial relationships that could be construed as a potential conflict of interest.

## Publisher's Note

All claims expressed in this article are solely those of the authors and do not necessarily represent those of their affiliated organizations, or those of the publisher, the editors and the reviewers. Any product that may be evaluated in this article, or claim that may be made by its manufacturer, is not guaranteed or endorsed by the publisher.

## References

[B1] Afshan S. Sharif A. (2016). Acceptance of mobile banking framework in Pakistan. Telemat. Inform. 33, 370–387. 10.1016/j.tele.2015.09.005

[B2] Ajzen I. Fishbein M. (1975). Belief, Attitude, Intention, and Behavior: an Introduction to Theory and Research. Reading, MA: Addison-Wesley pub. Co., 197.

[B3] Ajzen I. Fishbein M. (2005). The influence of attitudes on behavior, in The Handbook of Attitudes, ed Albarracín D. (Mahwah, NJ: Erlbaum), 173–221.

[B4] Akturan U. Tezcan N. (2012). Mobile banking adoption of the youth market. Mark. Intell. Plan. 30, 444–459. 10.1108/02634501211231928

[B5] Alalwan A. A. Dwivedi Y. K. Rana N. P. (2017). Factors influencing adoption of mobile banking by Jordanian bank customers: extending UTAUT2 with trust. Int. J. Inform. Manag. 37, 99–110. 10.1016/j.ijinfomgt.2017.01.002

[B6] Alalwan A. A. Rana N. P. Dwivedi Y. K. Lal B. Williams M. D. (2015). *Adoption of mobile banking in* Jordan: exploring demographic differences on customers' perceptions, in Open and Big Data Management and Innovation (New York, NY: Springer). 10.1007/978-3-319-25013-7_2

[B7] Alqatan S. Noor N. M. M. Man M. Mohemad R. (2016). An empirical study on success factors to enhance customer trust for mobile commerce in small and medium-sized tourism enterprises (SMTES) in Jordan. J. Theor. Appl. Inform. Technol. 83, 373–398.

[B8] Al-Somali S. A. Gholami R. Clegg B. (2009). An investigation into the acceptance of online banking in Saudi Arabia. Technovation 29, 130–141. 10.1016/j.technovation.2008.07.004

[B9] Baabdullah A. M. (2018). Consumer adoption of mobile social network games (M-SNGs) in Saudi Arabia: the role of social influence, hedonic motivation and trust. Technol. Soc. 53, 91–102. 10.1016/j.techsoc.2018.01.004

[B10] Baabdullah A. M. Alalwan A. A. Rana N. P. Kizgin H. Patil P. (2019). Consumer use of mobile banking (M-Banking) in Saudi Arabia: towards an integrated model. Int. J. Inform. Manag. 44, 38–52. 10.1016/j.ijinfomgt.2018.09.002

[B11] Baptista G. Oliveira T. (2015). Understanding mobile banking: the unified theory of acceptance and use of technology combined with cultural moderators. Comput. Hum. Behav. 50, 418–430. 10.1016/j.chb.2015.04.024

[B12] Bashir M. Strickland B. Bohr J. (2016). What motivates people to use Bitcoin?, in International Conference on Social Informatics (New York, NY: Springer), 347–367. 10.1007/978-3-319-47874-6_25

[B13] Baur A. W. Bühler J. Bick M. Bonorden C. S. (2015). Cryptocurrencies as a disruption? Empirical findings on user adoption and future potential of Bitcoin and Co. open and big data management and innovation, in Proceedings of the 14th IFIP WG 6.11 Conference on e-Business, e-Services and e-Society, I3E 2015. Proceedings: LNCS 9373, eds Janssen M.. 63–80. 10.1007/978-3-319-25013-7_6

[B14] Berentsen A. Schär F. (2018). A short introduction to the world of cryptocurrencies. Fed. Reserve Bank St. Louis Rev. First Q. 100, 1–16. 10.20955/r.2018.1-16

[B15] Bhattacherjee A. Sanford C. (2006). Influence processes for information technology acceptance: an elaboration likelihood model. MIS Q. J. Econ. 30, 805–825. 10.2307/25148755

[B16] Bock G.-W. Lee J. Kuan H. H. Kim J. H. (2012). The progression of online trust in the multi-channel retailer context and the role of product uncertainty. Decis. Support Syst. 53, 97–107. 10.1016/j.dss.2011.12.007

[B17] Chandra S. Srivastava S. C. Theng Y. L. (2010). Evaluating the role of trust in consumer adoption of mobile payment systems: an empirical analysis. Commun. Assoc. Inform. Syst. Res. Behav. Sci. 27, 29. 10.17705/1CAIS.02729

[B18] Chang H. H. Chen S. W. (2008). The impact of online store environment cues on purchase intention: trust and perceived risk as a mediator. Online Inform. Rev. 32, 818–841. 10.1108/14684520810923953

[B19] Chemingui H. Lallouna H. B. (2013). Resistance, motivations, trust and intention to use mobile financial services. Int. J. Bank Mark. 31, 574–592. 10.1108/IJBM-12-2012-0124

[B20] Chen Y.-H. Barnes S. (2007). Initial trust and online buyer behaviour. Ind. Manag. Data Syst. 107, 21–36. 10.1108/02635570710719034

[B21] Chong A. Y. L. (2013). Predicting m-commerce adoption determinants: a neural network approach. Exp. Syst. Appl. 40, 523–530. 10.1016/j.eswa.2012.07.068

[B22] Chopdar P. K. Korfiatis N. Sivakumar V. J. Lytras M. D. (2018). Mobile shopping apps adoption and perceived risks: a cross-country perspective utilizing the unified theory of acceptance and use of technology. Comput. Hum. Behav. 86, 109–128. 10.1016/j.chb.2018.04.017

[B23] Connolly A. J. Kick A. (2015). Bitcoin research past, present and future, in Proceedings of the 11th Annual USC Upstate Research Symposium.

[B24] Cooper B. Esser A. Allen M. (2019). The Use Cases of Central Bank Digital Currency for Financial Inclusion: A Case for Mobile Money. Washington, DC: The Center for Financial Regulation and Inclusion.

[B25] Dahlberg T. (2019). *What block*c*hain developers and users expect from virtual currency* regulations: a survey study. Inform. Polity 24, 453–467. 10.3233/IP-190145

[B26] Dennehy D. Sammon D. (2015). Trends in mobile payments research: a literature review. J. Innov. Manag. 3, 49–61. 10.24840/2183-0606_003.001_0006

[B27] Dwivedi Y. K. Rana N. P. Jeyaraj A. Clement M. Williams M. D. (2017). Reexamining the unified theory of acceptance and use of technology (UTAUT): towards a revised theoretical model. Inform. Syst. Front. 21, 1–16. 10.1007/s10796-017-9774-y

[B28] Ermakova T. Fabian B. Baumann A. Izmailov M. Krasnova H. (2016). Bitcoin: drivers and impediments. SSRN Elect. J. 2, 1–18. 10.2139/ssrn.3017190

[B29] Faqih K. M. Jaradat M. R. (2015). Assessing the moderating effect of gender differences and individualism-collectivism at individual-level on the adoption of mobile commerce technology: TAM3 perspective. J. Retail. Consum. Serv. 22, 37–52. 10.1016/j.jretconser.2014.09.006

[B30] Farah M. F. Hasni M. J. S. Abbas A. K. (2018). Mobile-banking adoption: empirical evidence from the banking sector in Pakistan. Int. J. Bank Mark. 36, 1386–1413. 10.1108/IJBM-10-2017-0215

[B31] Farell R. (2015). An Analysis of the Cryptocurrency Industry. Philadelphia, PA: University of Pennsylvania.

[B32] Fisher J. Burstein F. Lynch K. Lazarenko K. (2008). “Usability+ usefulness= trust”: an exploratory study of Australian health web sites. Internet Res. 18, 477–498. 10.1108/10662240810912747

[B33] Flavian C. Guinaliu M. Torres E. (2005). The influence of corporate image on consumer trust: a comparative analysis in traditional versus internet banking. Internet Res. 15, 447–470. 10.1108/10662240510615191

[B34] Folkinshteyn D. Lennon M. M. (2017). Braving bitcoin: a technology acceptance model analysis. J. Inform. Technol. Case Appl. Res. 18, 220–249. 10.1080/15228053.2016.1275242

[B35] Fornell C. Larcker D. F. (1981). Evaluating structural equation models with unobservable variables and measurement error. J. Mark. Res. 18, 39–47. 10.2307/3151312

[B36] Fuller M. A. Serva M. A. Benamati J. S. (2007). Seeing is believing: the transitory influence of reputation information on e-commerce trust and decision making. Decis. Sci. 38, 675–699. 10.1111/j.1540-5915.2007.00174.x

[B37] Gao X. Clark G. D. Lindqvist J. (2016). Of two minds, multiple addresses, and one ledger: characterizing opinions, knowledge, and perceptions of bitcoin across usrs and non-users, in Conference on Human Factors in Computing Systems—CHI '16, San Jose, CA, 1656–1668. 10.1145/2858036.2858049

[B38] Gefen D. (2000). E-commerce: the role of familiarity and trust. Omega 28, 725–737. 10.1016/S0305-0483(00)00021-9

[B39] Gefen D. Straub D. Boudreau M. C. (2003). Structural equation modeling and regression: guidelines for research practice. Commun. Assoc. Inform. Syst. 4, 56–79. 10.17705/1CAIS.00407

[B40] Gu J.-C. Lee S.-C. Suh Y.-H. (2009). Determinants of behavioral intention to mobile banking expert systems with applications. Exp. Syst. Appl. 36, 11605–11616. 10.1016/j.eswa.2009.03.024

[B41] Gunawan F. E. Novendra R. (2017). An analysis of bitcoin acceptance in Indonesia. ComTech Comput. Math. Eng. Appl. 8, 241. 10.21512/comtech.v8i4.3885

[B42] Guych N. Spyridou A. Simon Y. Jennet A. (2018). Factors influencing the intention to use cryptocurrency payments: an examination of blockchain economy, in TOURMAN 2018 Conference Proceedings (Rhodes: Greece), vol. 28, 303–310.

[B43] Gyu C. B. (2019). Influential factors on technology acceptance of mobile banking: focusing on mediating effects of trust. J. Distrib. Manag. Res. 22, 101–115.

[B44] Hair J. Anderson R. Tatham R. Black W. (1998). Multivariate Data Analysis with Readings. Englewood Cliffs, NJ: Prentice-Hall.

[B45] Hair J. F. Ringle C. M. Sarstedt M. (2011). PLS-SEM: indeed a silver bullet. J. Mark. Theory Pract. 19, 139–152. 10.2753/MTP1069-6679190202

[B46] Heijden H. V. D. (2004). User acceptance of hedonic information systems. MIS Q. 28, 695–704. 10.2307/25148660

[B47] Henry C. S. Huynh K. P. Nicholls G. (2018). Bitcoin awareness and usage in Canada. J. Dig. Bank. 2, 311–337.

[B48] Henseler J. Ringle C. M. Sinkovics R. R. (2009). The use of partial least squares path modeling in international marketing. Adv. Int. Mark. 20, 277–320. 10.1108/S1474-7979(2009)000002001424918859

[B49] Herero A. Martin H. S. Salmones M. G. (2017). Explaining the adoption of social networks sites for sharing user-generated content: a revision of the UTAUT2. Comput. Hum. Behav. 71, 209–217. 10.1016/j.chb.2017.02.007

[B50] Jiang L. A. Yang Z. Jun M. (2013). Measuring consumer perceptions of online shopping convenience. J. Serv. Manag. 24, 191–214. 10.1108/09564231311323962

[B51] Khan I. U. Hameed Z. Khan S. U. (2017). Understanding online banking adoption in a developing country: UTAUT2 with cultural moderators. J. Global Inform. Manag. 25, 43–65. 10.4018/JGIM.2017010103

[B52] Kim G. Shin B. Lee H. G. (2009). Understanding dynamics between initial trust and usage intentions of mobile banking. Inform. Syst. J. 19, 283–311. 10.1111/j.1365-2575.2007.00269.x

[B53] Kim K. K. Prabhakar B. (2004). *Initial trust and the adoption of B2C e-commerce: the case of internet* banking. ACM SIGMIS Database DATABASE Adv. Inform. Syst. 35, 50–64. 10.1145/1007965.1007970

[B54] Kim T. H. (2016). A study of digital currency cryptography for business marketing and finance security. Asia-Pacific J. Multimedia Serv. Converg. Art Hum. Sociol. 6, 365–376. 10.14257/AJMAHS.2016.01.42

[B55] Koenig-Lewis N. Palmer A. Moll A. (2010). Predicting young consumers' take up of mobile banking services. Int. J. Bank Mark. 28, 410–432. 10.1108/02652321011064917

[B56] Kraljić A. Pestek A. (2016). An application of Utaut2 model in exploring the impact of quality of technology on mobile internet. J. Econ. Bus. XIV, 66–77.

[B57] Kumpajaya A. Dhewanto W. (2015). The acceptance of Bitcoin in Indonesia: extending TAM with IDT. J. Bus. Manag. 4, 28–38.

[B58] Lallmahomed M. Z. Lallmahomed N. Lallmahomed G. M. (2017). Factors influencing the adoption of e-Government services in Mauritius. Telemat. Inform. 34, 57–72. 10.1016/j.tele.2017.01.003

[B59] Lee H. G. Han M. S. (2019). An empirical study on the consumer acceptance of internet primary bank: the application of UTAUT model. J. Bus. Edu. 33, 59–87. 10.34274/krabe.2019.33.1.003

[B60] Lee M. K. O. Turban E. (2001). A trust model for consumer internet shopping. Int. J. Elect. Commer. 6, 75–91. 10.1080/10864415.2001.11044227

[B61] Lee Y. C. Mu H. L. (2017). An application of fuzzy AHP and TOPSIS methodology for ranking the factors influencing FinTech adoption intention: a comparative study of China and Korea. J. Serv. Res. Stud. 7, 51–68. 10.18807/jsrs.2017.7.4.051

[B62] Li J. Wang J. Wangh S. Zhou Y. (2019). Mobile payment with alipay: an application of extended technology acceptance model. IEEE Access 7, 50380–50387. 10.1109/ACCESS.2019.2902905

[B63] Limayem M. Hirt S. G. Cheung C. M. K. (2007). How habit limits the predictive power of intentions: the case of IS continuance. MIS Q. 31, 705–737. 10.2307/25148817

[B64] Lin X. Chien S. W. Hung C. W. Chen S. C. Ruangkanjanases A. (2021). The impact of switching intention of telelearning in COVID-19 epidemic's era: the perspective of push-pull-mooring theory. Front. Psychol. 12, 639589. 10.3389/fpsyg.2021.639589 34393880 PMC8355365

[B65] Lin X. Suanpong K. Ruangkanjanases A. Lim Y. T. Chen S. C. (2022). Improving the sustainable usage intention of mobile payments: extended unified theory of acceptance and use of technology model combined with the information system success model and initial trust model. Front. Psychol. 12, 634911. 10.3389/fpsyg.2021.634911 35082707 PMC8784512

[B66] Lin X. Wu R. Lim Y. T. Han J. Chen S. C. (2019). Understanding the sustainable usage intention of mobile payment technology in Korea: crosscountries comparison of Chinese and Korean users. Sustainability 11, 5532–5555. 10.3390/su11195532

[B67] Lin X. Wu R. Z. (2021). An empirical study on the dairy product consumers' intention to adopt the food traceability's technology: push-pull-mooring model integrated by DandM ISS model and TPB With ITM. Front. Psychol. 11, 1–14. 10.3389/fpsyg.2020.612889 33519633 PMC7843444

[B68] Lin X. C. Chang S. C. Chou T. H. Chen S. C. Ruangkanjanases A. (2021). Consumers' intention to adopt blockchain food traceability technology towards organic food products. Int. J. Environ. Res. Public Health 18, 135. 10.3390/ijerph18030912 33494321 PMC7908134

[B69] Lu H. P. Su P. Y. J. (2009). Factors affecting purchase intention on mobile shopping web sites. Internet Res. 19, 442–458. 10.1108/10662240910981399

[B70] Lu M. T. Tzeng G. H. Cheng H. Hsu C. C. (2015). Exploring mobile banking services for user behavior in intention adoption: using new hybrid MADM model. Serv. Bus. 9, 541–565. 10.1007/s11628-014-0239-9

[B71] Mahfuz M. A. Khanam L. Hu W. (2016). The influence of culture on m-banking technology adoption: an integrative approaches of UTAUT2 and ITM, in Proceedings of the Portland International Conference on Management of Engineering and Technology (PICMET), 824–835. 10.1109/PICMET.2016.7806814

[B72] Mamonov S. Benbunan-Fich R. (2015). An empirical investigation of privacy breach perceptions among smartphone application users. Comput. Hum. Behav. 49, 427–436. 10.1016/j.chb.2015.03.019

[B73] Martin S. S. Catalan B. L. Ramon-Jeronimo M. A. (2013). Mobile shoppers: types, drivers, and impediments. J. Organ. Comput. Elect. Commun. 23, 350–371. 10.1080/10919392.2013.837793

[B74] Martins C. Oliveria T. Popovič A. (2014). Understanding the internet banking adoption: a unified theory of acceptance and use of technology and perceived risk application. Int. J. Inform. Manag. 34, 1–13. 10.1016/j.ijinfomgt.2013.06.002

[B75] McKnight D. H. Choudhury V. Kacmar C. (2002). The impact of initial consumer trust on intentions to transact with a web site: a trust building model. J. Strat. Inform. Syst. 11, 297–323. 10.1016/S0963-8687(02)00020-3

[B76] McKnight D. H. Cummings L. L. Chervany N. L. (1998). Initial trust formation in new organizational relationships. Acad. Manag. Rev. 23, 473–490. 10.5465/amr.1998.926622

[B77] McKnight D. H. Kacmar C. J. Choundhury V. (2004). Shifting factors and the ineffectiveness of third party assurance seals: a two-stage model of initial trust in a Web business. Elect. Mark. 14, 252–266. 10.1080/1019678042000245263

[B78] Mendoza G. G. Jung I. Kobayashi S. (2017). A review of empirical studies on MOOC acceptance: applying the unified theory of acceptance and use of technology. Int. J. Edu. Media Technol. 11, 15–24.

[B79] Mendoza-Tello J. C. Mora H. M. Pujol F. A. Lytras M. (2018). Social commerce as a driver to enhance trust and intention to use cryptocurrencies for electronic payments. IEEE Access 6, 50737–50751. 10.1109/ACCESS.2018.2869359

[B80] Nakamoto S. (2008). Bitcoin: A Peer-to-Peer Electronic Cash System. Available online: http://bitcoin.org/bitcoin.pdf (accessed August 30, 2019).

[B81] Pavlou P. David G. (2004). Building effective online marketplaces with institution-based trust. Inform. Syst. Res. 15, 37–59. 10.1287/isre.1040.0015

[B82] Pavlou P. Tan Y. H. Gefen D. (2003). The transitional role of institutional trust in online interorganizational relationships, in Proceedings of the 36th Hawaii International Conference on Systems Sciences, 10. 10.1109/HICSS.2003.1174574

[B83] Pavlou P. A. Fygenson M. (2006). Understanding and predicting electronic commerce adoption: an extension of the theory of planned behavior. MIS Q. 30, 115–143. 10.2307/25148720

[B84] PricewaterhouseCoopers (2019). The Rise of Central Bank Digital Currencies (CBDCs).

[B85] Queiroz M. M. Wamba S. F. (2019). Blockchain adoption challenges in supply chain: an empirical investigation of the main drivers in India and the USA. Int. J. Inform. Manag. 46, 70–82. 10.1016/j.ijinfomgt.2018.11.021

[B86] Raskin M. Yermack D. (2016). Digital Currencies, Decentralized Ledgers and the Future of Central Banking. Northampton, MA: Edward Elgar Publishing.

[B87] Reid J. Marion L. (2020). The Future of Payments: Part III. Digital Currencies: The Ultimate Hard Power Tool. Frankfurt: Deutsche Bank Research.

[B88] Rondan-Cataluna F. J. Arenas-Gaitan J. Ramirez-Correa P. E. (2015). A comparison of the different versions of popular technology acceptance models: a non-linear perspective. Kybernetes 44, 788–805. 10.1108/K-09-2014-0184

[B89] Schaper L. K. Pervan G. P. (2007). A model of information and communication technology acceptance and utilizations by occupational therapist. Int. J. Med. Inform. 76, 212–221. 10.1016/j.ijmedinf.2006.05.028 16828335

[B90] Seetharaman A. Saravanan A. S. Patwa N. Mehta J. (2017). Impact of bitcoin as a world currency. Account. Finance Res. 6, 230–246. 10.5430/afr.v6n2p230

[B91] Shahzad F. Xiu G. Wang J. Shahbaz M. (2018). An empirical investigation on the adoption of cryptocurrencies among the People of Mainland China. Technol. Soc. 55, 33–40. 10.1016/j.techsoc.2018.05.006

[B92] Shaw N. Sergueeva K. (2019). The non-monetary benefits of mobile commerce: extending UTAUT2 with perceived value. Int. J. Inform. Manag. 45, 44–55. 10.1016/j.ijinfomgt.2018.10.024

[B93] Shehhi A. A. Oudah M. Aung Z. (2014). IEEE International Conference on Industrial Engineering and Engineering Management, 1443–1447.33447104

[B94] Shi Y. Zhou S. (2020). Central bank digital currencies: towards a Chinese approach-design choices of digital currency electronic payment. JIBS Bus. Admin. 7, 1–76.

[B95] Silinskyte J. (2014). Understanding Bitcoin Adoption: Unified Theory of Acceptance and Use of Technology (UTAUT) Application. Leiden: Leiden Institute of Advanced Computer Sciences, University Leiden, 56.

[B96] Slade E. L. Dwivedi Y. K. Piercy N. C. Williams M. D. (2015). Modeling consumers' adoption intentions of remote mobile payments in the United Kingdom: extending UTAUT with innovativeness, risk, and trust. Psychol. Mark. 32, 860–873. 10.1002/mar.20823

[B97] Straub D. Gefen D. (2003). Managing user trust in B2C e-services. E-Serv. J. 2, 7–24. 10.2979/esj.2003.2.2.7

[B98] Sung H. (2019). A study on the determinants of intention to use mobile payment service: comparisons of gender and age group differences. Glob. Bus. Admin. Rev. 16, 115–142. 10.38115/asgba.2019.16.4.115

[B99] Tam C. Oliveira T. (2016). Understanding the impact of m-banking on individual performance: DeLone and McLean and TTF perspective. Comput. Hum. Behav. 61, 233–244. 10.1016/j.chb.2016.03.016

[B100] Tam C. Oliveira T. (2017). Literature review of mobile banking and individual performance. Int. J. Bank Mark. 35, 1042–1065. 10.1108/IJBM-09-2015-0143

[B101] Thompson R. L. Higgins C. A. Howell J. M. (1991). Personal computing: toward a conceptual model of utilization. MIS Q. 15, 124–143. 10.2307/249443

[B102] Thong J. Y. L. (2001). Resources constraints and information systems implementation in Singaporean small business. Omega 29, 143–156. 10.1016/S0305-0483(00)00035-9

[B103] Venkatesh V. Morris M. G. Davis G. B. Davis F. D. (2003). User acceptance of information technology: toward a unified view. MIS Q. 27, 425–478. 10.2307/30036540

[B104] Venkatesh V. Thong J. Y. L. Xu X. (2012). Consumer acceptance and use of information technology: extending the unified theory of acceptance and use of technology. MIS Q. 36, 157–178. 10.2307/41410412

[B105] Venkatesh V. Thong J. Y. L. Xu X. (2016). Unified theory of acceptance and use of technology: a synthesis and the road ahead. J. Assoc. Inform. Syst. 17, 328–376. 10.17705/1jais.00428

[B106] Verkijika S. F. (2018). Factors influencing the adoption of mobile commerce applications in Cameroon. Telemat. Inform. 35, 1665–1674. 10.1016/j.tele.2018.04.012

[B107] Walton A. Johnston K. (2018). Exploring perceptions of bitcoin adoption: the south african virtual community perspective. Interdiscip. J. Inform. Knowl. Manag. 13, 165–182. 10.28945/4080

[B108] Williams M. D. Rana N. P. Dwivedi Y. K. (2015). The unified theory of acceptance and use of technology (UTAUT): a literature review. J. Enterp. Inform. Manag. 28, 443–488. 10.1108/JEIM-09-2014-008832339928

[B109] Worasesthaphong T. (2015). Consumer trust in B2C e-commerces of the cluster fashion clothing and jewelry business. Rev. Integr. Bus. Econ. Res. 4, 231–240.

[B110] Wu R. Lee J.-H. (2017). The comparative study on third party mobile payment between UTAUT2 and TTF. J. Distrib. Sci. 15, 5–19. 10.15722/jds.15.11.201711.5

[B111] Xin H. Techatassanassoontorn A. A. Tan F. B. (2015). Antecedents of consumere trust in mobile payment adoption. J. Comput. Inform. Syst. 55, 1–10. 10.1080/08874417.2015.1164578133498863

[B112] Yeong Y. C. Kalid S. K. Sugathan S. K. (2019). Cryptocurrency acceptance: a case of Malaysia, in International conference on Recents Advancements in Engineering and Technology (ICRAET-18), 15th and 16th, March.

[B113] Yu C. S. (2012). Factors affecting individuals to adopt mobile banking: empirical evidence from the UTAUT model. J. Elect. Comm. Res. 13, 104–121.

[B114] Yu C. S. Asgarkhani M. (2015). An investigation of trust in e-banking: evidence from Taiwan and New Zealand Empirical Studies. Manag. Res. Rev. 38, 1267–1284. 10.1108/MRR-09-2014-0210

[B115] Zarifis A. Efthymiou L. Cheng X. Demetriou S. (2014). Consumer trust in digital currency enabled transactions, in Business Information Systems Workshops: BIS 2014 International Workshops, May 22–23, 241–254. 10.1007/978-3-319-11460-6_21

[B116] Zhang X. Guo X. Lai K. Yin C. Meng F. (2017). From offline healthcare to online health services: the role of offline healthcare satisfaction and habits. J. Elect. Comm. Res. 18, 138–154.

[B117] Zhou T. (2011). Understanding mobile internet continuance usage from the perspectives of UTAUT and flow. Inform. Develop. 27, 207–218. 10.1177/0266666911414596

[B118] Zhou T. (2012). Examining mobile banking user adoption from the perspectives of trust and flow experience. Inform. Technol. Manag. 13, 27–37. 10.1007/s10799-011-0111-8

